# The Millennium Development Goals: experiences, achievements and what's next

**DOI:** 10.3402/gha.v7.23695

**Published:** 2014-02-13

**Authors:** Marta Lomazzi, Bettina Borisch, Ulrich Laaser

**Affiliations:** 1 World Federation of Public Health Associations, c/o IGH/CMU, University of Geneva, Geneva, Switzerland; 2 Institute of Global Health, University of Geneva, Geneva, Switzerland; 3 Faculty of Health Sciences, University of Bielefeld, Bielefeld, Germany

**Keywords:** Millennium Development Goals, sustainable development, equity, education, accountability, governments, post-2015 agenda

## Abstract

The Millennium Development Goals (MDGs) are eight international development goals to be achieved by 2015 addressing poverty, hunger, maternal and child mortality, communicable disease, education, gender inequality, environmental damage and the global partnership. Most activities worldwide have focused on maternal and child health and communicable diseases, while less attention has been paid to environmental sustainability and the development of a global partnership. Up to now, several targets have been at least partially achieved: hunger reduction is on track, poverty has been reduced by half, living conditions of 200 million deprived people enhanced, maternal and child mortality as well as communicable diseases diminished and education improved. Nevertheless, some goals will not be met, particularly in the poorest regions, due to different challenges (e.g. the lack of synergies among the goals, the economic crisis, etc.). The post-2015 agenda is now under discussion. The new targets, whatever they will be called, should reflect today's political situation, health and environmental challenges, and an all-inclusive, intersectoral and accountable approach should be adopted.

The Millennium Development Goals (MDGs) are the most widely supported and comprehensive development goals the world has ever established. These eight goals and 18 targets provide a concrete framework for tackling poverty, hunger, maternal and child mortality, communicable disease, education, gender inequality, environmental damage and the global partnership for development (1) (Table 1).

**Table 1:** The eight Millennium Development Goals (MDGs)

MDG1	Eradicating extreme poverty and hunger
MDG2	Achieving universal primary education
MDG3	Promoting gender equality and empowering women
MDG4	Reducing child mortality rates
MDG5	Improving maternal health
MDG6	Combating HIV/AIDS, malaria and other diseases
MDG7	Ensuring environmental sustainability
MDG8	Developing a global partnership for development

These targets are both global and local, adapted to each country to meet specific needs. They provide a framework for the whole international community to work together towards a common goal. If these goals are achieved, world poverty will be reduced by half, millions of lives will be saved, and billions of people will benefit from the global economy in a more sustainable environment (2). Furthermore, the MDGs are inter-dependent and largely influence each other. For example, promoting gender equality and empowering women enables not only better conditions for women but also improved household management leading to better health and education for children and to higher income for the family.

The MDGs find their origins in development ideas and campaigns of the 1980s and 1990s; they were officially established following the Millennium Summit of the United Nations in 2000, as an output of the United Nations Millennium Declaration (3). All 189 United Nations member states agreed to achieve these goals on a voluntary basis by the year 2015. New global health initiatives (such as the Global Fund, the World Bank, the GAVI Alliance, etc.) and increased financial resources have advanced the opportunity to deliver MDG-related health programmes worldwide (4).

From 2000 on, important high-level meetings and summits have been organized to follow up with the progress in the MDGs and to define action plans for their achievement. In 2008, governments, foundations, businesses groups and civil society announced new commitments to meet the MDGs, during the high-level event at the UN Headquarters (5). Two years after, the 2010 MDG Summit concluded with the adoption of a global action plan – Keeping the Promise: United to Achieve the Millennium Development Goals – and announced a number of initiatives against poverty, hunger and disease, with a special focus on women's and children's health (6).

In 2013, participants in the Global MDG Conference underlined the importance of maintaining the momentum for accelerating progress to 2015, while taking lessons learned from the MDGs to be used in the development of the agenda of the next round of goals beyond 2015 (7).

## MDGs achievements and failures

To assure an appropriate monitoring and evaluation within and among countries and to conceive suitable policies and interventions, reliable, timely and internationally comparable data on the MDG indicators are of primary importance. They are also essential in encouraging funding and allocating aid effectively (8). Several methodologies and indicators (Table 2) have been developed to measure progress towards the MDGs, such as the MDG indicators website, the UN Data – and the UNICEF Portal (9–11). Moreover, progress towards MDG achievement can be tracked through the MDG Monitor, both globally and at the country level (12).

**Table 2:** Millennium Development Goals (MDGs) targets and indicators. Adapted from: http://www.unmillenniumproject.org/goals/gti.htm

MDGs	Targets	Indicators
MDG1	**Target 1**. Halve, between 1990 and 2015, the proportion of people whose income is less than $1 a day	**1**. Proportion of population below $1 (1993 PPP) per day (World Bank) **2**. Poverty gap ratio [incidence×depth of poverty] (World Bank) **3**. Share of poorest quintile in national consumption (World Bank)
	**Target 2**. Halve, between 1990 and 2015, the proportion of people who suffer from hunger	**4**. Prevalence of underweight children under five years of age (UNICEF–WHO) **5**. Proportion of population below minimum level of dietary energy consumption (FAO)
MDG2	**Target 3**. Ensure that, by 2015, children everywhere, boys and girls alike, will be able to complete a full course of primary schooling	**6**. Net enrolment ratio in primary education (UNESCO) **7**. Proportion of pupils starting grade 1 who reach grade 5 (UNESCO) **8**. Literacy rate of 15–24 year-olds (UNESCO)
MDG3	**Target 4**. Eliminate gender disparity in primary and secondary education, preferably by 2005, and in all levels of education no later than 2015	**9**. Ratio of girls to boys in primary, secondary and tertiary education (UNESCO) **10**. Ratio of literate women to men, 15–24 years old (UNESCO) **11**. Share of women in wage employment in the non-agricultural sector (ILO) **12**. Proportion of seats held by women in national parliament (IPU)
MDG4	**Target 5**. Reduce by two-thirds, between 1990 and 2015, the under-five mortality rate	**13**. Under-five mortality rate (UNICEF–WHO) **14**. Infant mortality rate (UNICEF–WHO) **15**. Proportion of 1 year-old children immunized against measles (UNICEF–WHO)
MDG5	**Target 6**. Reduce by three-quarters, between 1990 and 2015, the maternal mortality ratio	**16**. Maternal mortality ratio (UNICEF–WHO) **17**. Proportion of births attended by skilled health personnel (UNICEF–WHO)
MDG6	**Target 7**. Have halted by 2015 and begun to reverse the spread of HIV/AIDS	**18**. HIV prevalence among pregnant women aged 15–24 years (UNAIDS–WHO–UNICEF) **19**. Condom use rate of the contraceptive prevalence rate (UN Population Division) **19a**. Condom use at last high-risk sex (UNICEF–WHO) **19b**. Percentage of population aged 15–24 years with comprehensive correct knowledge of HIV/AIDS (UNICEF–WHO) **19c**. Contraceptive prevalence rate (UN Population Division) **20**. Ratio of school attendance of orphans to school attendance of non-orphans aged 10–14 years (UNICEF–UNAIDS–WHO)
	**Target 8**. Have halted by 2015 and begun to reverse the incidence of malaria and other major diseases	**21**. Prevalence and death rates associated with malaria (WHO) **22**. Proportion of population in malaria-risk areas using effective malaria prevention and treatment measures (UNICEF–WHO) **23**. Prevalence and death rates associated with tuberculosis (WHO) **24**. Proportion of tuberculosis cases detected and cured under DOTS (internationally recommended TB control strategy) (WHO)
MDG7	**Target 9**. Integrate the principles of sustainable development into country policies and programs and reverse the loss of environmental resources	**25**. Proportion of land area covered by forest (FAO) **26**. Ratio of area protected to maintain biological diversity to surface area (UNEP–WCMC) **27**. Energy use (kg oil equivalent) per $1 GDP (PPP) (TEA, World Bank)
		**28**. Carbon dioxide emissions per capita (UNFCCC, UNSD) and consumption of ozone-depleting CFCs (ODP tons) (UNEP-Ozone Secretariat) **29**. Proportion of population using solid fuels (WHO)
	**Target 10**. Halve, by 2015, the proportion of people without sustainable access to safe drinking water and basic sanitation	**30**. Proportion of population with sustainable access to an improved water source, urban and rural (UNICEF–WHO) **31**. Proportion of population with access to improved sanitation, urban and rural (UNICEF–WHO)
	**Target 11**. Have achieved by 2020 a significant improvement in the lives of at least 100 million slum dwellers	**32**. Proportion of households with access to secure tenure (UN–HABITAT)
MDG8	**Target 12**. Develop further an open, rule-based, predictable, non-discriminatory trading and financial system (includes a commitment to good governance, development and poverty reduction both nationally and internationally) **Target 13**. Address the special needs of the least developed countries [includes tariff- and quota-free access for least developed countries’ exports, enhanced program of debt relief for heavily indebted poor countries (HIPCs) and cancellation of official bilateral debt, and more generous official development assistance for countries committed to poverty reduction] **Target 14**. Address the special needs of landlocked developing countries and small island developing states (through the Program of Action for the Sustainable Development of Small Island Developing States and 22nd General Assembly provisions) **Target 15**. Deal comprehensively with the debt problems of developing countries through national and international measures in order to make debt sustainable in the long term	**Official development assistance (ODA)** **33**. Net ODA, total and to LDCs, as percentage of OECD/Development Assistance Committee (DAC) donors’ gross national income (GNI)(OECD) **34**. Proportion of total bilateral, sector-allocable ODA of OECD/DAC donors to basic social services (basic education, primary health care, nutrition, safe water and sanitation) (OECD) **35**. Proportion of bilateral ODA of OECD/DAC donors that is untied (OECD) **36**. ODA received in landlocked developing countries as a proportion of their GNIs (OECD) **37**. ODA received in small island developing States as proportion of their GNIs (OECD) **Market access** **38**. Proportion of total developed country imports (by value and excluding arms) from developing countries and from LDCs, admitted free of duty (UNCTAD, WTO, WB) **39**. Average tariffs imposed by developed countries on agricultural products and textiles and clothing from developing countries (UNCTAD, WTO, WB) **40**. Agricultural support estimate for OECD countries as percentage of their GDP (OECD) **41**. Proportion of ODA provided to help build trade capacity (OECD, WTO) Debt sustainability **42**. Total number of countries that have reached their Heavily Indebted Poor Countries Initiative (HIPC) decision points and number that have reached their HIPC completion points (cumulative) (IMF – World Bank) **43**. Debt relief committed under HIPC initiative (IMF-World Bank) **44**. Debt service as a percentage of exports of goods and services (IMF-World Bank)
Some of the indicators listed below are monitored separately for the least developed countries, Africa, landlocked developing countries and small island developing states		
	**Target 16**. In cooperation with developing countries, develop and implement strategies for decent and productive work for youth	**45**. Unemployment rate of young people aged 15–24 years, each sex and total (ILO)
	**Target 17**. In cooperation with pharmaceutical companies, provide access to affordable essential drugs in developing countries	**46**. Proportion of population with access to affordable essential drugs on a sustainable basis (WHO)
	**Target 18**. In cooperation with the private sector, make available the benefits of new technologies, especially information and communications technologies	**47**. Telephone lines and cellular subscribers per 100 population (ITU) **48**. Personal computers in use per 100 population and Internet users per 100 population (ITU)

Furthermore, there have been numerous consultations on the MDGs by various organizations. Some of the consultations and surveys have had an official character and others should be considered ‘private’ initiatives, by organizations such as non-governmental organizations (NGOs) and private foundations (13–18). More than a few official reports have tracked the global assessment of progress, based on those data (14, 19–21). Although considerable progress has been made, reliable data and statistics analyses remain poor, especially in many developing countries (8).

In the last 13 years, the MDGs have managed to focus world attention and global political consensus on the needs of the poorest and to achieve a significant change in the Official Development Assistance (ODA) commitments (22). They have provided a framework allowing countries to plan their social and economic development and donors to provide effective support at national and international level (8). Most activities worldwide have targeted MDGs 4, 5 and 6, focusing on maternal and child health (MCH) and communicable diseases, especially in the developing countries, while fewer initiatives have focused on MDGs 1, 2, 3 and 7, which are more difficult to influence (14). Some studies have underlined regional differences in the importance that is attributed to specific MDGs. For example, MDGs 4 and 5 have been considered most important in the African region, while MDGs 7 and 8 in the Western Pacific Region. Low-income countries have attached high relevance to MDG1 when compared to high-income countries (14, 23). Arab countries have not considered MDGs among the top priority for the policy makers, academia and social actors in general mainly due to ethnic, religious, political and social limitations (18).

The most recent UN report on progress towards the MDGs has highlighted several achievements in all health and education areas (21): the hunger reduction goal is on track; the target of decreasing extreme poverty by half has been met, as well as the goal of halving the proportion of people who lack steady access to drinking water; conditions for more than 200 million people living in favelas have been improved; significant achievements have been made in the fight against communicable diseases such as malaria and tuberculosis and child and maternal mortality have been reduced. Moreover, primary school admission of girls has equalled that of boys and developing countries experienced a reduced debt burden and an improved climate for trade (20, 21, 24, 25).

However, progress has been highly unequal. The reduction in global income poverty is mainly due to the rapid growth of a few countries in Asia, such as China, India, Indonesia and Vietnam. In many other countries, poverty reduction has been quite slow, or poverty has even increased (8). Sub-Saharan Africa remains the most underdeveloped region (8). Projections indicate that in 2015 more than 600 million people worldwide will still be using unsafe water sources, almost 1 billion will be living in very poor conditions, mothers will continue to die giving birth, and children will die from preventable diseases. Also, environmental sustainability remains a global challenge due to a fast decline of biodiversity and an increase in gas emissions. The goals of primary education and gender equality also remain unfulfilled, with broad negative consequences, given that achieving the MDGs deeply relies on education and women's empowerment. Moreover, there are severe inequalities that exist among populations, especially between rural and urban areas, or that affect marginalized people (20, 21). MDG8 remains one of the most challenging even if of primary importance for the achievement of all MDGs (8).

## Discussion on the effectiveness of the MDGs

As reported above, a major part of the MDGs has been at least partially accomplished and many countries are on the way to achieving the MDGs and trying to adopt a sustainable path (21). However, in spite of the general positive outputs, global targets will not be met in some regions, particularly sub-Saharan Africa and south Asia. Indeed, MDGs have encountered a range of common challenges (26).

First, they were not the product of a comprehensive analysis and prioritization of development needs and consequently were sometimes too narrowly focused. The inconsistent progress partly indicates a trend over time to focus on a subset of specific targets that were easier to achieve, implement and monitor (26). The untied nature of many goals has often affected the creation of the synergies that could arise across these targets and in particular between education, health, poverty and gender. Even if acceleration in one goal is likely to improve progress in others, these synergies are not always evident, and often vary across countries (26, 27).

Second, this framework has not afforded enough consideration to the potential impacts on environmental, social and economic dimensions. Environmental aspects are addressed under goal 7 but only some topics are covered, neglecting key issues for sustainable development. Most goals focus on the social dimension of development, e.g. MDGs 1, 2 and 6, addressing social problems such as hunger, education, equality, MCH and communicable diseases. However, these goals are also interconnected with environmental and economic factors. While some links are recognized (e.g. the importance of clean drinking water to health), others such as the maintenance of environmental resources or the quality of air are not. MDG8 addresses the implementation of sustainable development but does not consider new forms of financing, technology and capacity building (28).

Third, the issue of equity has represented one of the main challenges to face. A gender focus is clear only in MDGs 3 and 5, while it is missing throughout the other goals. MDG3 measures gender equality in education, employment and the proportion of women in national legislatures. MDG5 focuses on maternal mortality and access to reproductive health. This limited explicit inclusion in two MDGs is too narrow and clearly indicates that the gender issue and its dynamics have not yet been fully understood nor integrated in policy dialogues (26, 29). Improving equalities will require health system strengthening, associated with a political and social engagement to address all forms of discrimination (30).

Fourth, a lack of clear ownership and leadership internationally and nationally might have partially affected the achievement of the MDGs. Even if different countries scale up health services and make progress towards the MDGs at very different rates, we have mainly observed a trend to a global uniform approach. Rather than spreading specific technical interventions tested in one country on large scale, a more specific approach as well as the adoption of alternative models such as ‘learning by doing’ engaging key stakeholders and taking advantages from evidence-based data from pilot projects, might be adopted (26, 31). Furthermore, not only stakeholders but also public health professionals should be considered as key actors in the process. Indeed, it has been shown that understanding of MDGs among public health professionals was limited (14, 32). This general lack of information and awareness represents an important challenge. There is an absolute need for more elaborate publicity and awareness about the MDGs among key players if attaining the MDGs is to be a reality (33).

Fifth, achievement of the MDGs depends much on the fulfilment of MDG8 on global partnership. In his preface to the report, UN Secretary General Ban Ki-moon said, ‘At the just-concluded Rio+20 Conference, commitments were made on an ambitious sustainable development agenda. But to keep those pledges credible, we must deliver on previous commitments. As a world community, we must make rhetoric a reality and keep our promises to achieve the MDGs’ (8, 34). As reported above, almost 200 countries engaged themselves and provided substantial contributions to the cause. However, these commitments have not been always fully fulfilled. Engagement by governments (and donors in general) has been deeply affected by the global economic and financial crisis that has seriously undermined progress towards poverty reduction and MDGs achievement in general, from 2007 on. Furthermore, not only governments but also the private sector plays an essential role in the development of the global partnership. Up to now, more than half of the services used for MDGs have been provided by private sources and the role of the private sector is intended to be boosted in the next period. Thus, it is of primary importance that governments and the private sector work together to mobilize more resources to achieve the MDGs and counter the negative effect that the global financial crisis may have on the targets attained and future achievements (35, 36). Those investments should be sustainable over a long period and predictable, and innovative financing mechanisms might be taken in account (30).

Accountability must be an essential part of the framework. A few studies have underlined the problem of corruption in relation to the use of MDGs resources by governments and other organizations (14, 18, 37, 38). A health care system in a corrupt environment is weak and unstable, and it will be important for the post-2015 period to find solutions to address both the health and the governance aspects of the development agenda at the same time. Emerging governance models can allow larger citizen participation, ownership and influence, as well as intersectoral action. The participation of civil society and its accountability is essential for a strong new policy development and implementation process (30, 39, 40).

Last but not least, goal measurement is often too narrow, or might not identify a clear means of delivery (26). A lack of scientifically valid data on some MDGs, such as MDGs 5 and 6, did not allow the improvement achieved to be measured adequately or to be compared with a baseline (41). Government reports have sometimes been criticized as false and government-driven, leading to a lack of confidence into the official reporting systems (14, 18, 37, 38). More and better data are definitely needed, especially relating to the poorest and most vulnerable people. However, even the limited data systems available in some developing countries have allowed the making of assessable investments in education, health, essential infrastructure and environment (42).

## The post-2015 agenda

Despite the positive achievements attained, many see the health MDGs as ‘unfinished business’. Indeed, MDGs have not fully addressed the large concept of development included in the Millennium Declaration, which comprises human rights, equity, democracy and governance (30). A post-2015 slowdown must be avoided. The Millennium Declaration is still valid and the work should be finalized. To fully address this, the new targets, whatever they will be called, should follow the new political situation and include the emerging countries. The framework adopted for the MDGs should be adapted to today's needs: new power, new countries, new groups of the poor and new partnerships. The notion of good health is progressing, shifting towards a people-centred approach to create and preserve good health and well-being rather than preventing and treating diseases. Health is now a societal issue of the global community and should be considered as a global good (43). Health systems should be able to adapt to more complex expectations and new health and environmental challenges. New ways are emerging to improve health: new technologies allow unique access to information and enable civil society worldwide to be connected and take part in the decision-making process. In this way, marginalized people can also be integrated in the debate (30). A strong emphasis might be placed on the importance of learning and sharing knowledge and experiences of best practices (30).

The post-2015 health agenda should also include specific sustainable health-related targets as well as take an all-inclusive approach to preserving people's health for the entire lifespan. As a first step, the current MDGs targets should be achieved and new targets should be adopted for addressing, e.g. the burden of non-communicable diseases (NCDs – such as cardiovascular diseases, cancers, chronic respiratory diseases and diabetes), sexual education, aging, mental illness and other emerging health challenges such as human mobility and refugees (13, 30). Equity and education should be considered as the base of health and incorporated in all targets. The links between health and sustainable development goals (SDGs), as underlined in the Rio +20 report, need to be strengthened with a rigorous framework and the new agenda should adopt a social determinant of health approach (13, 44). Indeed, improving people's health and quality of life cannot be achieved by focusing only on the health sector, but requires action to address the wider socioeconomic issues that influence how people live and get sick, including risk factors, services availability and accessibility, etc. These conditions depend on the distribution of resources and power at local and global levels. An integrated ‘health-in-all-policies’ approach involving different sectors linked to governance, environment, education, employment, social security, food, housing, water, transport and energy are necessary in order to address the complexity of health inequities (30, 45–47). Global health diplomacy is nowadays focusing on the development of such a framework, thus incorporating health as a part of all policies or, on the other hand, starting from health to drive policies to protect national security, free trade and economic advancement. Health should be perceived as an investment and not only as a cost (44, 48).

Accountability remains of primary importance. On one hand, better data will be required to allow transparency, proper evaluation and improvements. On the other hand, governments’ engagement and partnership dynamics between all actors should be improved and adapted to the new socio-political context.

The north–south division is no longer applicable; NCDs such as obesity are affecting all, independently of their country income, with a negative impact not only on human well-being but also on national productivity.

Any future health goal must be universally relevant; however, targets and indicators must be adaptable to a country's health priorities and needs and regional differences should be considered (14, 30).

The role of governments internationally and at the local level, including in areas such as health workforce recruitment and supply and production of products for health (e.g. vaccines), should be improved. On one side, low and lower-middle income countries (23) should be able to mobilize local resources and improve in-country productivity as well as bring innovations and solutions that are more suitable for emerging countries. On the other side, rich countries should contribute more to the UN system. MDGs were agreed on a voluntary base by governments; the new goals should be norms for global governance and to reinforce the concept of the ‘right to health’. These targets should be global social contracts between governances and societies, and the concept of social responsibility, lacking for the MDGs, should be included.

A more efficient partnership among the different agencies could be envisaged, reducing to a few effective organizations the numbers of agencies involved. The dynamic between the actors should change: public–private partnerships are assuming more and more importance. The private for-profit (commercial companies) and not-for-profit [e.g. Bill and Melinda Gates Foundation (49)] sector is the only one that can afford the huge cost associated with this framework: no country, even the very rich, can replace this role. Moreover, the private sector should not be considered only as a donor but be embedded in the path, taking advantages of the capabilities offered by the sector.

## The new goals: picking and choosing

Everyone who has a cause wants a goal: however, to be successful the new goals should be limited to a few. Moreover, we are experiencing a sort of ‘goals anxiety’ due to a spasmodic search for fast-defined, effective and universal goals able to include all major issues. A careful consideration of all aspects in the due time would most probably lead to better definition of the goals.

Most of the discussions are focusing on two types of comprehensive goals for health: universal health coverage (UHC) and healthy life expectancy (HALE).

UHC and access could represent a successful model to achieve health goals and improve people's health at large (30). Margaret Chan, Director General of the WHO, has stated that ‘Universal Health Coverage is the single most powerful concept that public health has to offer’ and the Rio+20 conference recognized that UHC has the potential to reduce inequalities, improve economic growth and strengthen social organization (50). To achieve UHC, health services and infrastructures as well as coverage with financial risk protection should be guaranteed to everyone (51).

Maximizing HALE could be the other health goal. To achieve this aim, we should be able to ensure that people not only survive but enjoy good health throughout their lifespan (46, 51).

Both goals are linked and interconnected: an increase in HALE can be measured as an indicator and outcome of progress towards UHC and the UHC can be considered as the mechanism to improve HALE (51). Both UHC and HALE are interesting targets but their measurement will be challenging.

Debates about post-MDG targets and linkages with SDGs are now on going with in-country and thematic consultations, including, e.g. a UN Task Team, a post-2015 high-level panel established by the UN Secretary General, society consultations through social media, an Open Working Group provided by the UNSG in consultation with governments, etc. (44, 52–54).

Regardless of which overarching targets will be selected, the goals must be translated into measurable indicators; accountability and regular reviews of progress should be easy to perform, to share and to be understood by governments and the general public (13). A multisectoral approach will be essential, integrating the social determinants of health and with a main focus on equity, education and poverty reduction.

## Summary and conclusions

The MDGs have focused world attention on the needs of the poorest and driven countries and donors commitments to the achievement of common goals.

Even if a major part of the MDGs has been at least partially accomplished, many see the MDGs as ‘unfinished business’. A post-2015 slowdown must be prevented. A new round of goals is now under definition, aiming at fully addressing the large concept of sustainable development included in the Millennium Declaration. A new framework, an intersectoral approach and strong commitments by governments and donors would be of primary importance to define effective goals and translate them into reality.
